# Perioperative hemoglobin decrement as an independent risk of poor early graft function in kidney transplantation

**DOI:** 10.1186/s13104-020-05262-4

**Published:** 2020-09-05

**Authors:** Arpa Chutipongtanate, Arpakorn Kantain, Atiporn Inksathit, Surasak Kantachuvesiri, Vasant Sumethkul, Siriwan Jirasiritham, Sopon Jirasiritham, Somchai Chutipongtanate

**Affiliations:** 1 Department of Anesthesiology, Faculty of Medicine Ramathibodi Hospital, Mahidol University, Bangkok, Thailand; 2 Department for Clinical Epidemiology and Biostatistics, Faculty of Medicine Ramathibodi Hospital, Mahidol University, Bangkok, Thailand; 3 Division of Nephrology, Department of Medicine, Faculty of Medicine Ramathibodi Hospital, Mahidol University, Bangkok, Thailand; 4 Excellence Center of Organ Transplantation, Faculty of Medicine Ramathibodi Hospital, Mahidol University, Bangkok, Thailand; 5 Division of Vascular and Organ Transplant Surgery, Department of Surgery, Faculty of Medicine Ramathibodi Hospital, Mahidol University, Bangkok, Thailand; 6 Pediatric Translational Research Unit, Department of Pediatrics, Faculty of Medicine Ramathibodi Hospital, Mahidol University, 270 Rama VI Rd, Ratchathewi, Bangkok, 10400 Thailand

**Keywords:** Hemoglobin, Kidney transplantation, Perioperative medicine, Poor early graft function

## Abstract

**Objective:**

Perioperative change of hemoglobin concentration (Hb) was associated with acute kidney injury in patients who underwent non-cardiac surgery, but has never been investigated in kidney transplant patients. This study aimed to observe the effects of perioperative Hb change on early graft function in kidney transplant recipients.

**Results:**

A total of 269 kidney transplant patients were enrolled, of whom 98 (36.4%) developed poor early graft function (PEGF), and 171 (63.6%) had immediate graft function. Comparing two groups, patients with PEGF had a greater decremental change of Hb (−1.60 [−2.38,−0.83] vs. −0.70 [−1.35,0.20] g/dL, respectively; *p* < 0.001). A Hb cut-point of −1.35 g/dL was obtained from ROC analysis. Multivariate analysis showed that perioperative Hb decrement greater than 1.35 g/dL was an independent risk of PEGF (adjusted OR of 2.52, 95% CI 1.11–5.72; *p* = 0.026). Subgroup analysis revealed deceased donor kidney transplant (DDKT; n = 126) (adjusted OR of 2.89, 95% CI 1.11–7.55; *p* = 0.029), but not living donor kidney transplantation (LDKT; n = 143) (adjusted OR of 1.68, 95% CI 0.23–12.15; *p* = 0.606), was influenced by the perioperative Hb decrement. In conclusion, this study suggests that decremental change in perioperative Hb greater than 1.35 g/dL may serve as a modifiable factor of PEGF in DDKT.

## Introduction

Quality of early graft function in kidney transplant patients is an important predictor for long-term graft outcome. Early graft function can be divided into delayed graft function (DGF), slow graft function (SGF), and immediate graft function (IGF). The incidence of DGF varies between 10 and 54.2%, and is associated with an increased risk of acute rejection and subsequent poor long-term graft survival [1–7]. However, its definition is not consistent, leading to conflicting data [6, 8]. Slow graft function (SGF) is defined as an intermediate condition between DGF and IGF [9, 10], and represents part of the spectrum of graft injury [10]. Patients with SGF also had poorer long-term graft outcome than patients with IGF [9–11]. Since both SGF and DGF determined a similarly negative impact on patient and graft survival [10–12], poor early graft function (PEGF) that covered the overall spectrum of graft dysfunction has been initially applied when evaluating the significance of early graft function [12, 13]. Long-term graft survival, acute rejection-free survival, and 1-year renal function in living donor kidney transplant patients who developed PEGF were significantly lower than those with IGF [12–14].

Risk factors of PEGF have still been under investigation. Factors being associated with PEGF were recipients BMI, pre-transplant dialysis, advanced donor age, and prolonged warm ischemic time (WIT) [12–14]. Since DGF is a subset of PEGF, risk factors such as deceased donor type, prolonged cold ischemic time (CIT), higher rate of HLA mismatch, and/or higher panel reactive antibody level (PRA) [2–4, 15], may also contribute to PEGF. It is desirable identifying modifiable factors of PEGF as a measure to improve long-term graft outcomes.

We hypothesized that perioperative change in Hb may serve as a modifiable risk factor of PEGF. Two lines of evidence supported this hypothesis. First, Abou-Jaoude et al., reported that pre-operative hemoglobin levels, either too high or too low, both affected early and long-term graft outcomes in kidney transplant patients [16]. Second, Walsh et al., demonstrated that even small decrements in perioperative hemoglobin concentration were strongly associated with acute kidney injury in non-cardiac surgical patients with normal pre-operative renal function [17]. Therefore, patients with kidney transplant should be even more vulnerable to changes of hemoglobin levels during the perioperative period, and acute kidney injury would manifest as poor early graft function in this context.

The aim of this study was to investigate perioperative Hb change in patients with PEGF as compared to IGF. The association between PEGF and perioperative Hb change was further analysed by univariate and multivariate analyses. Other variables were also systematically evaluated to identify the additional risk of PEGF.

## Main text

### Methods

#### Sample population

This study was approved by the Ethical Clearance Committee on Human Right Related to Research involving Human Subjects, Faculty of Medicine Ramathibodi Hospital, Mahidol University (Protocol ID 03-57-39). Informed consent was waived because of retrospective nature of the study. Patient information was anonymized and de-identified before analysis. Data were retrospectively collected from medical records of patients ≥ 18 years of age who underwent first kidney transplant at Ramathibodi Hospital, Thailand, during January 1, 2011 and December 31, 2013. Requirements were at least 1 pre and 1 post-operative hemoglobin measurement within 24-h of surgery; patients with post-operative acute rejection were excluded. Demographic data were recorded as shown in Table 1. The reason and common conditions of deceased donors included brain death due to traffic-related trauma (52%), cerebrovascular accident (31%), fall and other trauma (10%), and other causes (7%), i.e., cardiovascular diseases, primary brain tumors, and asphyxia. All patients received immunosuppressive therapy, including a calcineurin inhibitor (tacrolimus or cyclosporine), antiproliferative agents (mycophenolate mofetil or azathioprine) and prednisolone.

**Table 1:** Characteristics of recipients, donors and intraoperative variables

Variables	PEGF (n = 98)	IGF (n = 171)	*P*-value
Recipients			
Age (year), mean ± SD	42.7 ± 10.8	41.4 ± 11.7	0.393
Gender, male	62 (63.3)	102 (59.6)	0.604
BMI (kg/m^2^), mean ± SD	22.4 ± 4.2	21.6 ± 3.8	0.087
Comorbid			0.125
Smoking	20 (20.4)	30 (17.5)	
Diabetes mellitus	5 (5.1)	28 (16.4)	
Hypertension	89 (90.8)	155 (90.6)	
Coronary artery disease	3 (3.1)	9 (5.3)	
Cerebrovascular disease	0 (0.0)	2 (1.2)	
Causes of ESRD			0.020
Diabetes mellitus	2 (2.0)	16 (9.4)	
Hypertension	2 (2.0)	2 (1.2)	
Renal diseases	20 (20.4)	70 (40.9)	
Congenital	1 (1.0)	6 (3.5)	
Unknown/others	70 (71.4)	91 (53.2)	
Dialysis modality			0.165
Hemodialysis	87 (88.8)	143 (83.6)	
Peritoneal dialysis	9 (9.2)	21 (12.3)	
Baseline creatinine (mg/dL), mean ± SD	9.48 ± 3.7	8.18 ± 3.7	0.007
Baseline pre-transplant hemoglobin (g/dL), median [IQR]	11.8 [10.7,13.2]	11.4 [10.1,12.6]	0.700
Postoperative hemoglobin (g/dL), median [IQR]	10.4 [9.4, 11.1]	10.6 [9.6, 11.7]	0.150
Positive anti-CMV	94/95 (98.9)	163/166 (98.2)	0.520
Pretransplant PRA (%), median [IQR]	0 [0, 53]	0 [0, 0]	0.003
Pretransplant blood transfusion	31 (31.6)	50 (29.2)	0.721
Donors			
Age (year), mean ± SD	43.9 ± 14.5	39.0 ± 11.2	0.002
Gender, male	76 (77.6)	83 (48.5)	< 0.001
HLA-A + B + DR mismatch, median [IQR]	2 [2, 3]	3 [2, 4]	0.012
Deceased donor	87 (88.7)	39 (22.8)	< 0.001
WIT (min), median [IQR]	45 [38, 55]	39 [32, 50]	0.004
CIT (min), median [IQR]	1205 [909, 1378]	27 [18, 72]	< 0.001
Intraoperative			
Intravenous fluid (L), median [IQR]	3.6 [3.0, 4.2]	3.5 [2.9, 4.1]	0.737
Blood transfusion	10 (10.2)	23 (13.5)	0.435
EBL (mL), median [IQR]	400 [300, 600]	300 [200, 400]	< 0.001
Need for vasopressor	21 (21.4)	45 (26.3)	0.378
Perioperative			
Perioperative Hb change (g/dL), median [IQR]	−1.6 [−2.38, −0.83]	−0.7 [−1.35, 0.20]	< 0.001
Percentage of Hb decrement (%), median [IQR]	12.6 [7.0, 17.5]	5.9 [−1.7, 11.9]	< 0.001

If not indicated otherwise: n (%)

### Definitions

IGF was defined as a serum creatinine < 3 mg/dL on postoperative day 5 [12]. SGF was defined as a serum creatinine ≥ 3 mg/dL on postoperative day 5 without the need of dialysis during the first postoperative week [12]. DGF was defined as the need for dialysis within the first postoperative week, which was the most frequent used definition [6, 12]. PEGF included both DGF and SGF [12–14], thus covered the overall spectrum of graft dysfunction.

### Data analysis

Patients were divided into two groups; those with IGF and those with PEGF, according to the definitions above. The change in perioperative Hb was calculated by subtracting the last known pre-operative from the lowest within-24-h post-operative Hb. The percentage of Hb decrement was calculated by 100*[pre-transplant Hb − postoperative Hb]/pre-transplant Hb. To evaluate the association between perioperative Hb change and PEGF, a cut-point was defined using receiver operating characteristic (ROC) curve. In case sensitivity and specificity needed to be traded off, the cut-point values with 75–80% specificity were screened to select the value with the best-possible sensitivity and the highest AUC. To identify the other PEGF risk factors, all significant variables from descriptive analysis were included into logistic regression analysis.

### Statistical analysis

Statistical analysis was performed by Excel and R packages. Quantitative data were presented as percentage, proportion, mean ± SD or median and interquartile range [IQR] as appropriate. Chi-square or Fischer’s exact test were used for categorical variables, while Student’s t-test or Mann–Whitney U-tests were used for continuous variables. The confidence intervals of the AUC was calculated by the DeLong method. Univariate and multivariate analysis were used to estimate crude OR and adjusted OR with 95% CI. *P*-value of < 0.05 was considered as statistical significance.

### Results

Initially altogether 319 kidney transplant recipients were assessed for eligibility and 280 recipients were included (Additional file 1: Figure S1). Eleven patients were excluded due to acute rejection post-operatively (Additional file 1: Figure S1). Of the remaining 269 patients, 164 were males; mean age was 41.8 years (range 18–63). There were 171 patients with IGF (63.6%) and 98 patients with PEGF (36.4%); characteristics of the groups are described in detail in Table 1. The PEGF group comprised 68 DGF and 30 SGF patients. Almost all recipient related parameters were similar between the groups, including baseline pre-transplant hemoglobin and postoperative hemoglobin. Differences were the cause of end-stage renal disease (ESRD), recipient baseline creatinine, and pre-transplant PRA. In contrast, almost all variables of donor and intraoperative factors had statistical difference between PEGF and IGF groups (as shown in Table 1). The estimated blood loss (EBL) in the PEGF group was significantly greater than in the IGF group (400 [300, 600] vs. 300 [200, 400] mL; *p* < 0.001), whereas both groups received intraoperative blood transfusions comparably (10.2 vs. 13.5%; *p* = 0.435). Note that the need for vasopressor (a reflective indicator of perioperative hypotension) had no statistically different between groups. All variables with statistically significant between IGF and PEGF groups were submitted into logistic regression analysis. Although WIT was not significant in univariate analysis, this variable was also included into multivariate analysis because of its potential influence on perioperative Hb changes and being the risk of PEGF in the previous study [13].

The change (decrease) of Hb in patients with PEGF was significantly more pronounced as in patients with IGF (−1.60 [−2.38, 0.83] vs. −0.70 [−1.35, 0.20] g/dL; *p* < 0.001) (Additional file 2: Figure S2A). The percentage of Hb decrement, which covered the confounding effect of anemic/non-anemic Hb change, exhibited a similar result (12.6 [7.0, 17.5] vs. 5.9 [−1.7, 11.9] %; p < 0.001) (Additional file 2: Figure S2B). ROC curves were generated to define the optimal cut-points of both Hb parameters (Additional file 3: Figure S3). Within a range of 75–80% specificity, the cut-point values of −1.35 g/dL perioperative Hb change and 12.5% Hb decrement provided better sensitivity and AUC than the others (Additional file 4: Table S1; full details in Additional file 5: Table S2). These cut-points were therefore applied for further association analysis. Note that pre-transplant and postoperative hemoglobin levels as simple variables were excluded from univariate analysis due to their limited performances in ROC analysis (Additional file 3: Figure S3).

Univariate analysis showed nearly all evaluated variables were significantly associated with PEGF, including a decremental change in perioperative Hb > 1.35 g/dL (crude OR = 3.62, 95% CI 2.13–6.14; *p* < 0.001) and the percentage of Hb decrement > 12.5% (crude OR = 3.33, 95% CI 1.97–5.65; *p* < 0.001) (Table 2). Since both parameters exhibited an excellent correlation (Additional file 2: Figure S2C), the stronger predictor from univariate analysis (perioperative Hb decrement > 1.35 g/dL) was served as the representative for further evaluation by multivariate analysis. All significant variables from univariate analysis were submitted to multivariate analysis to estimated their association with PEGF and also weigh the influence of perioperative Hb. Multivariate analysis showed perioperative Hb decrement > 1.35 g/dL was an independent risk of PEGF (adjusted OR = 2.52, 95% CI 1.11–5.72; *p* = 0.026) (Table 3). Of other significant variables from univariate analysis, only CIT was proved as the risk of PEGF by multivariate analysis (adjusted OR = 9.77, 95%CI 1.58–60.54; *p* = 0.014) (Table 3).

**Table 2:** Univariate analysis to identify the candidate risks of PEGF

Variables	PEGF n (%)	IGF n (%)	Crude OR (95% CI)	*P*-value
Cause of ESRD				
Unknown	70 (71.4)	91 (53.2)	2.19 (1.29–3.74)	0.004
Known	28 (28.6)	80 (46.8)		
Recipient baseline creatinine (mg/dL)				
>8.5	54 (56.8)	64 (39.0)	2.06 (1.23–3.44)	0.007
≤8.5	41 (43.2)	100 (60.9)		
Pretransplant PRA (%)				
>50	24 (25.0)	15 (8.9)	3.4 (1.68–6.87)	<0.001
≤50	72 (75.0)	153 (91.1)		
Donor type				
Deceased	87 (88.8)	39 (22.8)	26.77 (13.33–56.65)	<0.001
Living	11 (11.2)	132 (77.2)		
Donor age (year)				
>55	21 (21.9)	13 (7.8)	3.29 (1.57–6.94)	0.002
≤55	75 (78.1)	153 (92.2)		
Donor gender				
Female	22 (22.4)	88 (51.5)	0.27 (0.15–0.47)	<0.001
Male	76 (77.6)	83 (48.5)		
HLA-A+B+DR mismatch				
>2	75 (76.5)	144 (84.2)	0.61 (0.33–1.14)	0.143
≤2	23 (13.5)	27 (15.8)		
WIT (min)				
>45	42 (43.8)	54 (32.5)	1.59 (0.95–2.68)	0.084
≤45	54 (56.3)	111 (66.8)		
CIT (h)				
>12	84 (85.7)	33 (19.3)	25.09 (13.44–53.09)	<0.001
≤12	14 (14.3)	138 (80.7)		
EBL (mL)				
>500	28 (28.6)	23 (13.5)	2.57 (1.38–4.79)	0.003
≤500	70 (71.4)	148 (86.5)		
Perioperative Hb decrement (g/dL)				
>1.35	53 (54.1)	42 (24.6)	3.62 (2.13–6.14)	<0.001
≤1.35	45 (45.9)	129 (75.4)		
Percentage of Hb decrement (%)				
>12.50	51 (52.0)	42 (24.6)	3.33 (1.97–5.65)	<0.001
≤12.50	47 (48.0)	129 (75.4)

**Table 3:** Multivariate analysis to identify PEGF-associated factors

Variables	Whole cohort (n=269)		Deceased donor (n=126)		Living donor (n=143)	
	Adjusted OR (95% CI)	*P*-value	Adjusted OR (95% CI)	*P*-value	Adjusted OR (95% CI)	*P*-value
Unknown cause of ESRD	1.84 (0.81–4.19)	0.148	1.85 (0.68–4.98)	0.227	1.30 (0.25–6.82)	0.753
Recipient baseline creatinine >8.5 mg/dL	1.83 (0.83–4.03)	0.135	1.57 (0.61–4.05)	0.349	3.18 (0.64–15.77)	0.157
Pretransplant PRA >50%	1.77 (0.62–5.07)	0.288	1.79 (0.55–5.83)	0.332	3.55 (0.27–46.51)	0.335
Deceased donor type	2.96 (0.41–21.37)	0.282	nd	nd	nd	nd
Donor age >55 years	1.37 (0.45–4.20)	0.538	1.61 (0.46–5.62)	0.452	nd	nd
Female donor	0.89 (0.35–2.23)	0.798	1.03 (0.31–3.42)	0.968	0.47 (0.09–2.37)	0.359
WIT >45 min	0.77 (0.35–1.69)	0.798	1.06 (0.42–2.69)	0.897	0.18 (0.01–2.37)	0.176
CIT >12 h	9.77 (1.58–60.54)	0.014	11.49 (1.89–69.77)	0.008	nd	nd
EBL >500 mL	1.71 (0.65–4.49)	0.276	1.73 (0.54–5.59)	0.360	2.86 (0.41–19.95)	0.289
Perioperative Hb decrement >1.35 g/dL	2.52 (1.11–5.72)	0.026	2.89 (1.11–7.55)	0.029	1.68 (0.23–12.15)	0.606

*nd* no data and not applicable

Although donor type (deceased donor vs. living donor) was not statistically associated with PEGF in multivariate analysis, differences in donor physiology should still be taken into consideration. Subgroup analysis was therefore performed to elucidate the influence of perioperative Hb decrement on PEGF based upon donor types. The result showed perioperative Hb decrement > 1.35 g/dL was the risk of PEGF in deceased donor (adjusted OR = 2.89, 95% CI 1.11–7.55; *p* = 0.029), but not in living donor (adjusted OR = 1.68, 95% CI 0.23–12.15; *p* = 0.606) (Table 3). Of note, the dataset used for univariate and multivariate analyses was provided in Additional file 6: Table S3. Lastly, the dose–effect relationship of perioperative Hb decrement on the occurrence of PEGF in DDKT patients was demonstrated in Additional file 7: Figure S4.

### Discussion

This study demonstrated that decremental change in perioperative Hb greater than 1.35 g/dL served as an independent risk of PEGF development in deceased donor kidney transplantation, but not living donors. Deceased donor kidney grafts, which had prolonged ischemic injury, were apparently more vulnerable to changes of hemoglobin levels than kidneys from living donors. Reduction of hemoglobin at a significant level may reduce oxygen delivery to glomeruli and renal tubules, subsequently aggravate graft function impairment in deceased donor kidney transplant. Changes in Hb may represent perioperative insult, particularly intraoperative bleeding in conjunction with hemodilution due to excessive intravenous fluid administration. Our findings support the hypothesis of Guedes-Marques, et al. [18], that perioperative changes in hemoglobin concentration may serve as a novel modifiable factor of PEGF in patients undergoing deceased donor kidney transplant.

Several known risk factors of DGF were re-evaluated to clarify their association with PEGF in our study. As expected, CIT was confirmed as the strongest risk of PEGF. Deceased donor type was usually collinear with CIT. It was not surprising that deceased donor type failed to show association with PEGF in multivariate analysis, where CIT had the strongest influence. The previously identified risks of DGF (i.e., older donor, female donor, and higher PRA) [2–4, 15], and PEGF (i.e., recipient BMI, pre-transplant dialysis, and prolonged WIT) [13, 14] were not statistically confirmed as PEGF-associated factors when multivariate analysis was applied, probably due to the relative small sample size, or their associations were negated under the strong influence of CIT and/or perioperative Hb decrement.

The advantage of blood transfusion has to be weighted with its adverse reactions. The goal of blood transfusion is to maintain adequate oxygen delivery to tissue. Currently, the optimal point is still controversial, especially in ESRD patients who have variations in baseline hemoglobin and compensation mechanism. Many studies emphasized the risk of blood transfusion in kidney transplant patients, particularly allosensitization [19]. Meticulous surgical hemostasis and appropriate perioperative measures to lessen blood loss are the most appropriate approach to prevent perioperative Hb decrement. Nonetheless, in the situation that blood transfusion is unavoidable, our data suggested a target hemoglobin level should be raised within 0–1.35 g/dL of pre-operative hemoglobin levels. These practices might be benefits to reduce PEGF incidence and improve long-term graft outcomes. Further prospective study is undoubtedly required to prove this hypothesis.

## Limitations

Several limitations included the lack of external validation, the small sample size, and the possible omission of confounders. The selected cut-off was based on the clinical intuition, while a data-driven estimation of the cut-off e.g., Youden-index might provide the better cut-off. Variations in treatment protocols i.e., the threshold for blood transfusion, accepted blood pressure and/or surgical technique might affect the outcomes. Also, there were several missing data due to retrospective nature of the study. Finally, long-term graft outcome was not evaluated in this study.

## Supplementary information

**Additional file 1: Figure S1.** A flow chart describing the data collection procedure based on inclusion and exclusion criteria, and the excluded patient due to missing data.

**Additional file 2: Figure S2.** Comparison between patients with poor early graft function (PEGF; n = 98) and immediate graft function (IGF; n = 171). **A**. Perioperative hemoglobin change was defined by the difference between post-operative and pre-operative hemoglobin concentrations (details in “**Materials and Methods**” section). **B**. The percentage of hemoglobin decrement was calculated by 100*[pre-transplant hemoglobin – postoperative hemoglobin]/pre-transplant hemoglobin. **C**. A scatter plot showed a linear correlation between both parameters. The dashed blue line represented the selected cut-points based on Receiver Operating Characteristics (as shown in **Table S1**). Red color dots represent patients with PEGF, whereas grey color dots represent patients with IGF. Visually, both hemoglobin decrement measures associated with an increasing trend of PEGF. Data were presented as median and interquartile range [IQR]. *P*-value < 0.05 was considered as statistically significant.

**Additional file 3: Figure S3.** The area under the ROC curve of various Hb parameters. The selected cut-off values for each Hb parameter which produced the specificity of 75–80% were shown in **Table S1**, while the full range of the sensitivity and specificity of all thresholds were provided in **Table S2**.

**Additional file 4: Table S1.** Area under the curve (AUC), sensitivity and specificity of the selected cut-point of perioperative Hb change, the percentage of Hb decrement, baseline pre-transplant hemoglobin and postoperative hemoglobin levels.

**Additional file 5: Table S2.** Sensitivities and specificities of all threshold of various Hb parameters including perioperative Hb change, the percentage of Hb decrement, baseline pre-transplant hemoglobin and postoperative hemoglobin levels.

**Additional file 6: Table S3.** The dataset used for univariate and multivariate analyses (n = 269 samples).

**Additional file 7: Figure S4.** The dose–effect relationship of perioperative Hb decrement on the occurrence of PEGF in DDKT patients (n = 126). The influence of perioperative Hb decrement, at each cut-off, was adjusted by the cause of ESRD, recipient baseline creatinine, Pretransplant PRA, donor age, donor gender, WIT, CIT and EBL as presented in Table 3.

## Data Availability

The dataset used or analysed during this study are included in this published article and its supplementary information files.

## Abbreviations

Hb: Hemoglobin
CIT: Cold ischemic time
DDKT: Deceased donor kidney transplant
DGF: Delayed graft function
EBL: Estimated blood loss
ESRD: End-stage renal disease
IGF: Immediate graft function
IQR: Interquartile range
LDKT: Living donor kidney transplantation
PEGF: Poor early graft function
PRA: Panel reactive antibody level
ROC: Receiver operating characteristic
SGF: Slow graft function
WIT: Warm ischemic time
