# Supplementary figures and images for: Perioperative hemoglobin decrement as an independent risk of poor early graft function in kidney transplantation
Source: BMC Res Notes. 2020 Sep 5;13:417. doi: 10.1186/s13104-020-05262-4 (PMC7487588; doi:10.1186/s13104-020-05262-4)

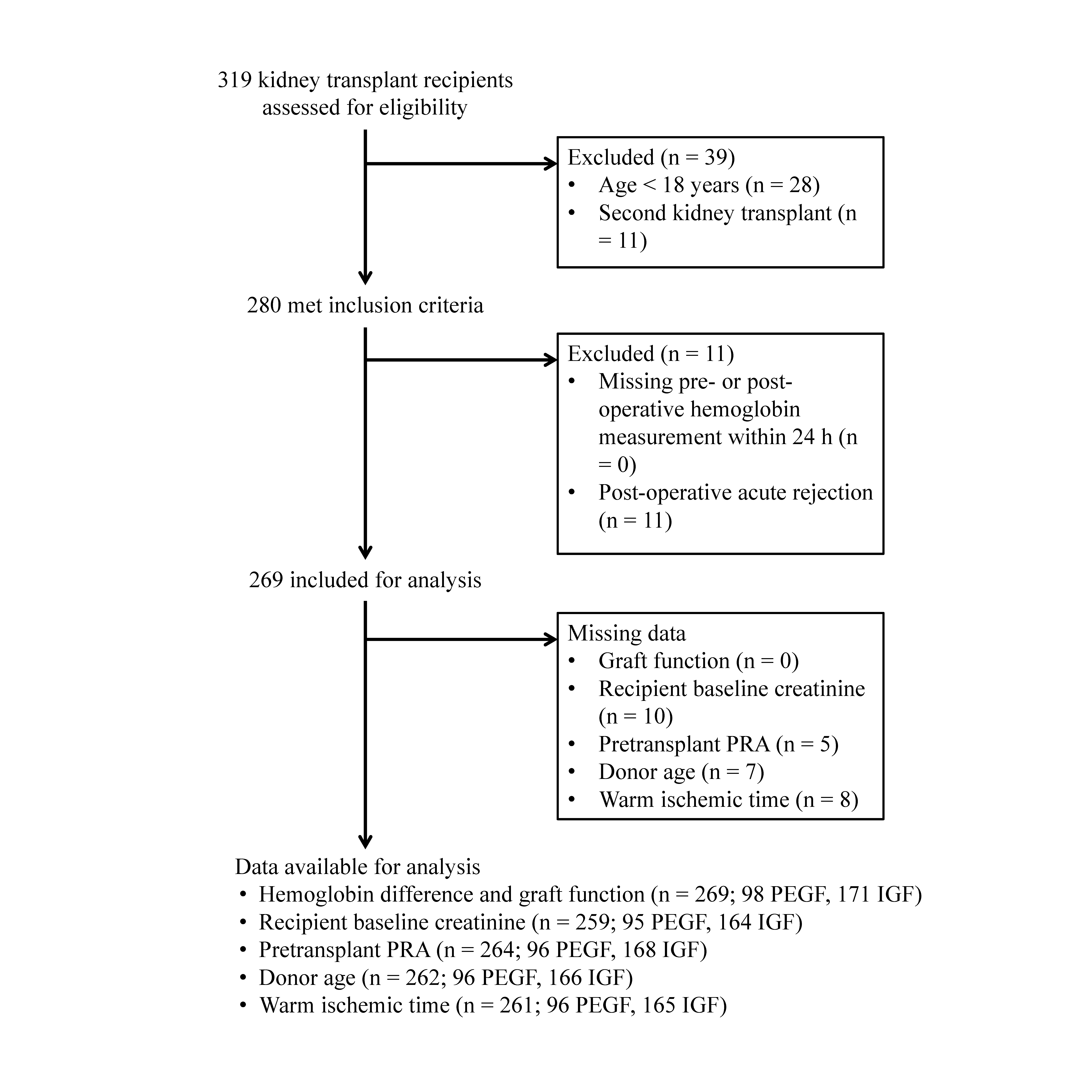

Supplement: Supplementary file 1 — Additional file 1: Figure S1. A flow chart describing the data collection procedure based on inclusion and exclusion criteria, and the excluded patient due to missing data. [file 13104_2020_5262_MOESM1_ESM.tiff]

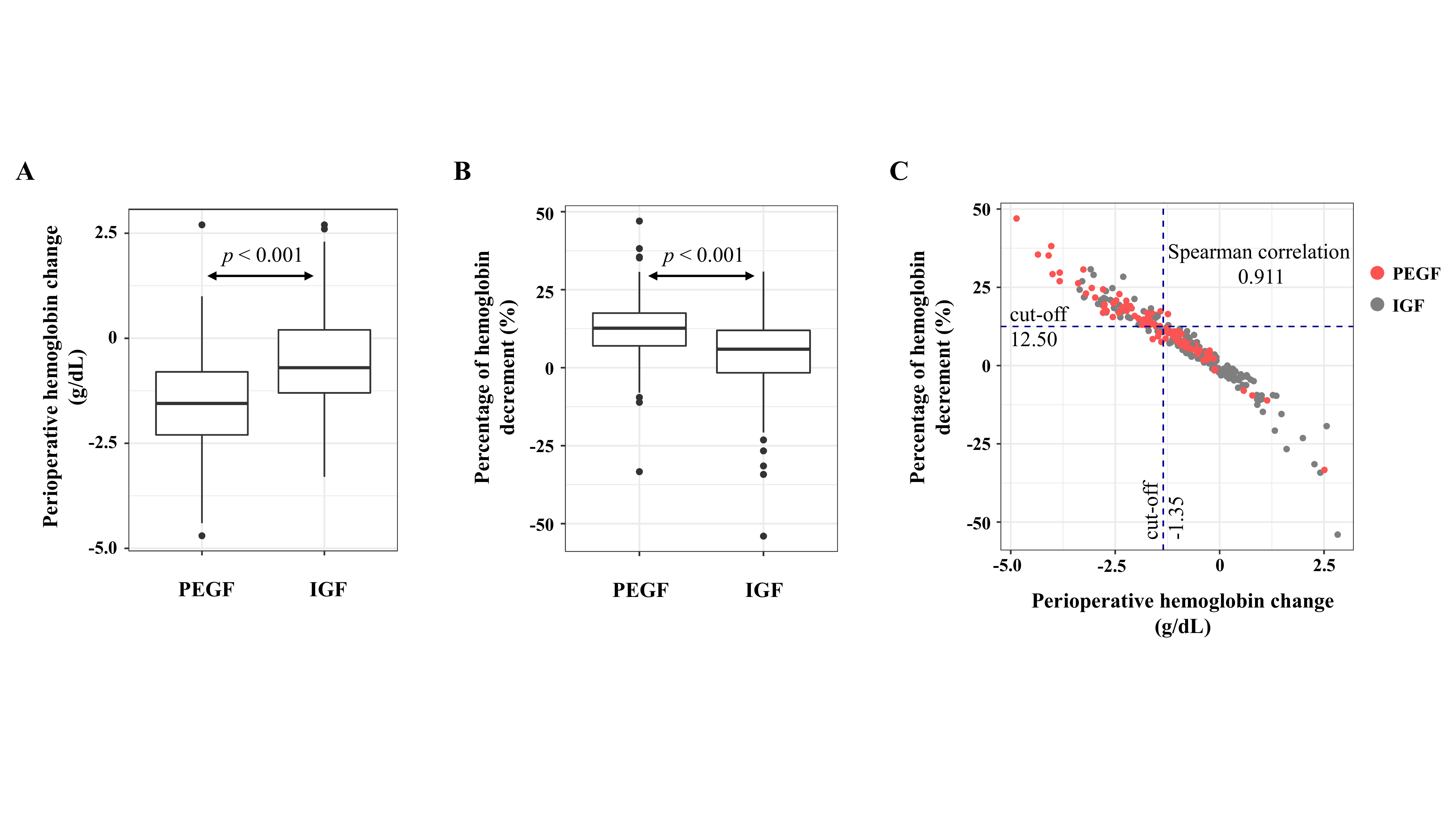

Supplement: Supplementary file 2 — Additional file 2: Figure S2. Comparison between patients with poor early graft function (PEGF; n = 98) and immediate graft function (IGF; n = 171). A. Perioperative hemoglobin change was defined by the difference between post-operative and pre-operative hemoglobin concentrations (details in “Materials and Methods” section). B. The percentage of hemoglobin decrement was calculated by 100*[pre-transplant hemoglobin – postoperative hemoglobin]/pre-transplant hemoglobin. C. A scatter plot showed a linear correlation between both parameters. The dashed blue line represented the selected cut-points based on Receiver Operating Characteristics (as shown in Table S1). Red color dots represent patients with PEGF, whereas grey color dots represent patients with IGF. Visually, both hemoglobin decrement measures associated with an increasing trend of PEGF. Data were presented as median and interquartile range [IQR]. P-value < 0.05 was considered as statistically significant. [file 13104_2020_5262_MOESM2_ESM.tif]

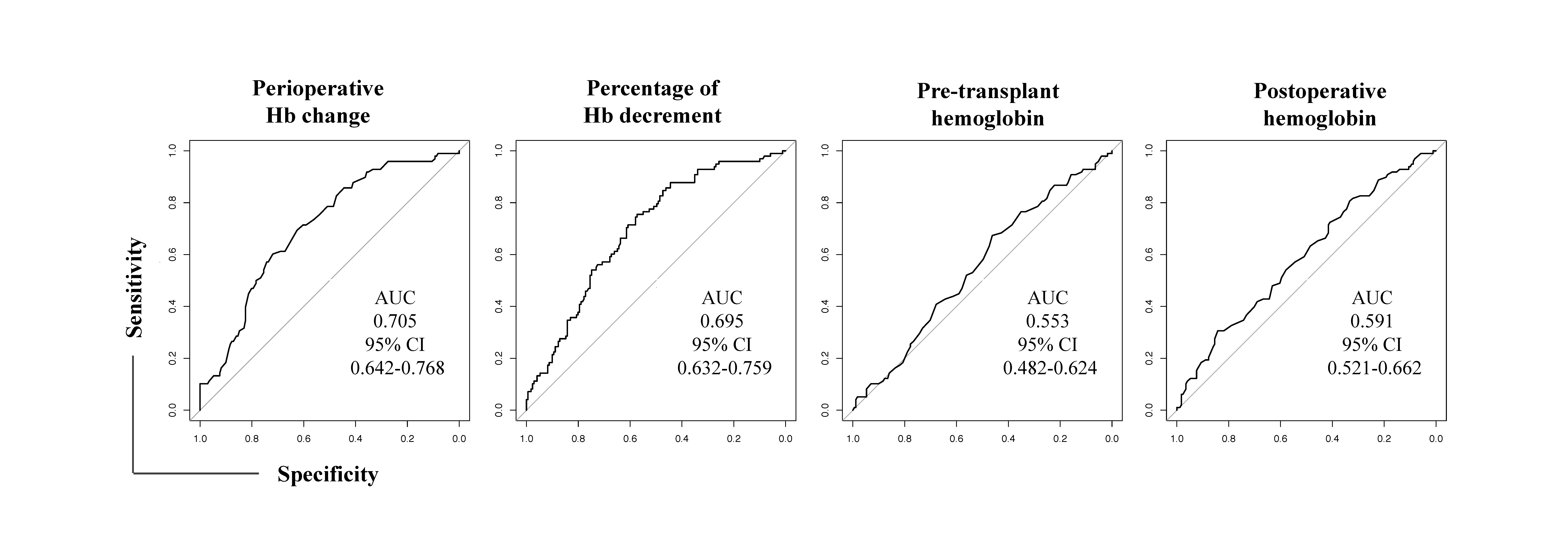

Supplement: Supplementary file 3 — Additional file 3: Figure S3. The area under the ROC curve of various Hb parameters. The selected cut-off values for each Hb parameter which produced the specificity of 75–80% were shown in Table S1, while the full range of the sensitivity and specificity of all thresholds were provided in Table S2. [file 13104_2020_5262_MOESM3_ESM.tiff]

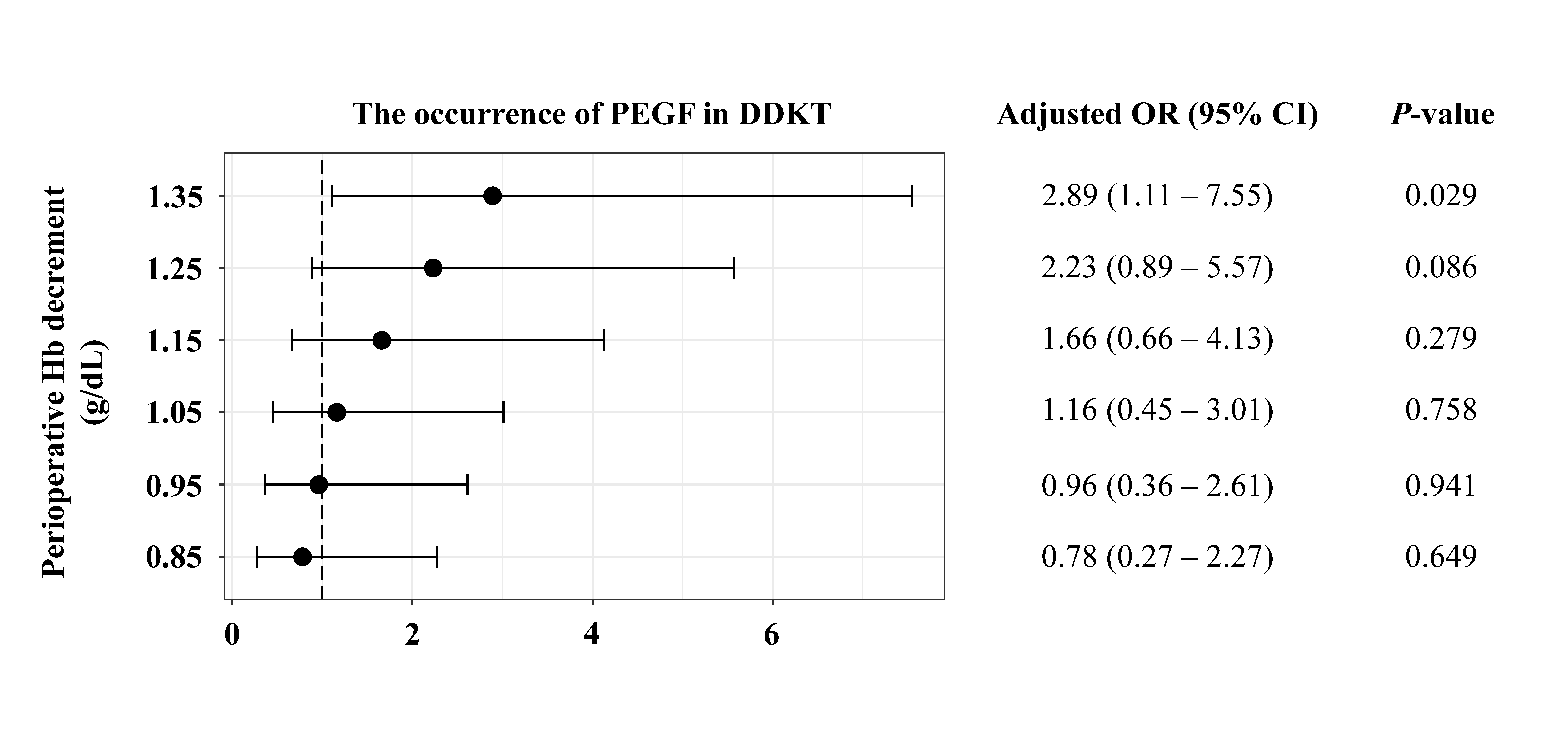

Supplement: Supplementary file 7 — Additional file 7: Figure S4. The dose–effect relationship of perioperative Hb decrement on the occurrence of PEGF in DDKT patients (n = 126). The influence of perioperative Hb decrement, at each cut-off, was adjusted by the cause of ESRD, recipient baseline creatinine, Pretransplant PRA, donor age, donor gender, WIT, CIT and EBL as presented in Table 3. [file 13104_2020_5262_MOESM7_ESM.tiff]
