# Supplementary material for: Perioperative hemoglobin decrement as an independent risk of poor early graft function in kidney transplantation
Source: BMC Res Notes. 2020 Sep 5;13:417. doi: 10.1186/s13104-020-05262-4 (PMC7487588; doi:10.1186/s13104-020-05262-4)
Supplement: Supplementary file 4 — Additional file 4: Table S1. Area under the curve (AUC), sensitivity and specificity of the selected cut-point of perioperative Hb change, the percentage of Hb decrement, baseline pre-transplant hemoglobin and postoperative hemoglobin levels. [file 13104_2020_5262_MOESM4_ESM.docx]

**Table S1.** Area under the curve (AUC), sensitivity and specificity of the selected cut-point of perioperative Hb change, the percentage of Hb decrement, baseline pre-transplant hemoglobin and postoperative hemoglobin levels.

|  | **AUC**  **(95% CI)** | | ***P*-value** | | **Sensitivity (%)** | | **Specificity (%)** | |
| --- | --- | --- | --- | --- | --- | --- | --- | --- |
| **Perioperative Hb change (g/dL)** |  | | | | | | | |
| -1.35 | 0.648  (0.578-0.717) | | <0.001 | | 54.1 | | 75.4 | |
| -1.45 | 0.638  (0.568-0.709) | | <0.001 | | 51.0 | | 76.6 | |
| -1.55 | 0.642  (0.571-0.712) | | <0.001 | | 50.0 | | 78.4 | |
| -1.65 | 0.635  (0.564-0.706) | | <0.001 | | 46.9 | | 80.1 | |
| **Percentage of Hb decrement (%)** |  | | | | | | | |
| 12.50 | 0.637  (0.578-0.697) | | <0.001 | | 52.0 | | 75.4 | |
| 13.00 | 0.615  (0.556-0.674) | | <0.001 | | 46.9 | | 76.0 | |
| 13.50 | 0.605  (0.547-0.664) | | <0.001 | | 43.9 | | 77.2 | |
| 14.00 | 0.599  (0.541-0.657) | | <0.001 | | 40.8 | | 78.9 | |
| 14.50 | 0.597  (0.539-0.654) | | <0.001 | | 39.8 | | 79.5 | |
| 15.00 | 0.584  (0.528-0.641) | | 0.002 | | 36.7 | | 80.1 | |
| **Pre-transplant hemoglobin (g/dL)** | |  | | | | | | |
| 9.00 | | 0.517  (0.482-0.551) | | 0.358 | | 92.9 | | 10.5 |
| 10.00 | | 0.543  (0.495-0.591) | | 0.092 | | 84.7 | | 24.0 |
| 11.00 | | 0.549  (0.490-0.609) | | 0.108 | | 68.4 | | 41.5 |
| 12.00 | | 0.536  (0.475-0.597) | | 0.243 | | 42.9 | | 64.3 |
| 13.00 | | 0.565  (0.513-0.616) | | 0.010 | | 27.6 | | 85.4 |
| **Postoperative hemoglobin (g/dL)** | |  | | | | | | |
| 9.00 | | 0.500  (0.454-0.546) | | 0.991 | | 16.3 | | 83.6 |
| 10.00 | | 0.543  (0.483-0.603) | | 0.153 | | 40.8 | | 67.8 |
| 11.00 | | 0.555  (0.496-0.615) | | 0.073 | | 68.4 | | 42.7 |
| 12.00 | | 0.539  (0.493-0.585) | | 0.111 | | 86.7 | | 21.1 |
| 13.00 | | 0.499  (0.467-0.531) | | 0.969 | | 92.9 | | 7.0 |
